# Platelet-Rich Fibrin in Surgical Wound Healing of Medication-Related Osteonecrosis of the Jaw: A Pilot Clinical Study

**DOI:** 10.3390/ijms27083654

**Published:** 2026-04-20

**Authors:** Aleksy Nowak, Aleksandra Rudzka, Piotr Skrzypczak, Krzysztof Osmola, Marzena Liliana Wyganowska

**Affiliations:** 1 Department of Maxillofacial Surgery, Poznan University of Medical Sciences, 49 Przybyszewskiego St., 60-355 Poznan, Poland; 2 Department of Dental Surgery, Periodontology and Oral Mucosa Diseases, Poznan University of Medical Sciences, 70, Bukowska St., 60-812 Poznan, Poland; 3 Doctoral School, Poznan University of Medical Sciences, 61-701 Poznan, Poland; 4 Orthodontic Clinic at University Center of Dentistry and Specialist Medicine, Poznan University of Medical Sciences, 60-812 Poznan, Poland; 5 Department of Thoracic Surgery, Poznan University of Medical Sciences, Szamarzewskiego 62 St., 60-569 Poznan, Poland

**Keywords:** MRONJ, platelet-rich fibrin, osteonecrosis of the jaw, bisphosphonates, oral surgery, maxillofacial surgery

## Abstract

Medication-related osteonecrosis of the jaw (MRONJ) represents a major clinical challenge for oral and maxillofacial surgery departments as well as dental practices. With increasing life expectancy and the more frequent use of medications associated with osteonecrosis, the incidence of MRONJ continues to rise. To date, there are no uniform treatment standards with scientifically proven effectiveness for this condition. To evaluate the impact of platelet-rich fibrin (PRF) on the outcomes of MRONJ treatment and to identify factors that may influence the effectiveness of PRF therapy, we conducted a comparative prospective study including 22 patients divided into two groups: patients treated with PRF and patients treated without PRF. PRF was prepared according to the PRF Duo Quattro Process protocol for PRF (Nice, France). The study was registered at ClinicalTrials.gov (NCT07464678). The following parameters were assessed: age, smoking status, gender, lesion location, body mass index (BMI), C-reactive protein (CRP) concentration, pain intensity, presence or absence of fistulas, soft tissue healing and radiological findings. Patients were evaluated preoperatively and postoperatively at 14 days, 6 weeks, and 6 months. The study demonstrated a reduction in pain after surgery among patients treated with PRF. In addition, the use of PRF resulted in improved healing outcomes in patients with elevated CRP. Higher BMI was associated with poorer therapeutic response to PRF. Improvements in soft tissue healing and disease stage were observed in the PRF group; however, these differences did not reach statistical significance. All findings should be interpreted with caution due to the limited sample size. There is still no standardized treatment for MRONJ. The use of platelet-rich fibrin as an inexpensive and safe adjunctive therapy may provide clinical benefits for patients, particularly through a significant reduction in pain. Further large-scale, multicenter studies are required to confirm these findings.

## 1. Introduction

Medication-related osteonecrosis of the jaw (MRONJ) currently represents a growing clinical problem. For more than 20 years since this disease entity was first described, no effective treatment method has been developed, despite numerous studies and scientific publications [1,2].

According to the definition of the American Association of Oral and Maxillofacial Surgeons (AAOMS), MRONJ is defined as exposed bone of the jaws that fails to heal within 8 weeks in a patient receiving medications known to be associated with this condition, in the absence of a history of radiotherapy to the head and neck region [3].

The pathogenesis of MRONJ is complex and multifactorial. Initially, bisphosphonate therapy was considered the primary causative factor, which led to the original term BRONJ (bisphosphonate-related osteonecrosis of the jaw). Clinical similarities between MRONJ and the historically described “phossy jaw” [4] observed in workers employed in phosphorus-processing factories, a chemical element that constitutes a component of bisphosphonate molecules, were also noted. Over time, similar necrotic lesions were observed in patients treated with denosumab, kinase inhibitors or glucocorticosteroids, which resulted in an expansion of the definition and the introduction of the term MRONJ [4].

Bisphosphonates affect osteoclast function by binding to hydroxyapatite molecules within bone tissue. Once internalized by osteoclasts, bisphosphonates inhibit their activity, shorten their lifespan, and limit the recruitment of new osteoclasts [5,6]. The result of this process is inhibition of bone remodeling, which, on the one hand, slows the progression of osteoporosis and limits the development of metastatic disease but, on the other hand, impairs the physiological regeneration of bone tissue [7]. Consequently, the bone microenvironment becomes less favorable for tumor cells while simultaneously becoming more susceptible to the development of necrosis, which constitutes one of the most frequently proposed mechanisms underlying the pathogenesis of MRONJ.

Osteoclasts inhibited by bisphosphonates lose the ability to respond appropriately to inflammatory processes, thereby facilitating their more rapid spread. In this context, inflammation may be triggered by trauma or infection originating from infected surrounding tissues. This leads to a subsequent increase in the concentration of inflammatory mediators, such as interleukins, and to the recruitment of immune system cells in an attempt to control the infection. Persistent inflammation combined with impaired osteoclast function ultimately results in the development of necrosis. In patients with MRONJ elevation of inflammatory markers may be observed in blood tests due to the ongoing inflammatory process [8].

Inhibition of bone cell function is not the only pathogenic factor in this condition. Certain drugs, such as zoledronic acid, also play a significant role in inducing necrotic processes by affecting the vascularization of oral soft tissues. This drug also inhibits vascular endothelial growth factor (VEGF) activity, leading to impaired angiogenesis, prolonged healing and consequently an increased risk of MRONJ treatment failure [9]. Reduced vascularization of the affected area weakens the body’s defense mechanisms, and the higher incidence of MRONJ in the mandible, which is less well vascularized than the maxilla, appears to support this association.

Numerous studies have identified infection and trauma as major risk factors for MRONJ. Among 60–80% of patients with MRONJ, a tooth extraction was performed prior to the onset of necrotic symptoms [10,11,12]. Additional risk factors for MRONJ include tobacco smoking, poor oral hygiene, malignant disease, poorly controlled diabetes mellitus, autoimmune diseases, HIV (Human Immunodeficiency Virus) infection, advanced age, hypothyroidism and genetic factors [3,4,10,13].

Clinical manifestations of MRONJ include pain, halitosis (fetor ex ore), the formation of intraoral and extraoral fistulas, sinusitis, sensory disturbances, and pathological fractures. Several classification systems describing the severity of MRONJ have been proposed. For the purposes of the present study, the classification developed by the AAOMS [3] was used, which categorizes patients based on clinical and radiological findings using a four-stage scale.

Stage 0—clinical symptoms without radiological changes or with nonspecific radiological findings; among patients at this stage, approximately 50% will progress to a higher stage.

Stage I—visible exposed necrotic bone or an intraoral fistula probing to bone; asymptomatic patients without signs of infection.

Stage II—visible exposed necrotic bone or an intraoral fistula probing to bone; symptomatic patients with signs of infection.

Stage III—visible exposed necrotic bone or an intraoral fistula probing to bone; symptomatic patients with signs of infection and one of the following: necrosis extending beyond the alveolar bone/alveolar process, pathological fracture, extraoral fistula, oroantral fistula or osteolysis of the sinus floor or the inferior border of the mandible.

Reduced expression of VEGF and other factors responsible for tissue repair processes in patients with MRONJ provided the rationale for the use of platelet-rich fibrin (PRF) as an adjunctive treatment modality.

PRF is one of the plasma fractions obtained through the process of blood centrifugation. It contains platelets, lymphocytes, stem cells, monocytes, neutrophils and a fibrin network. Platelets present in PRF release numerous bioactive factors, including von Willebrand factor (vWF), P-selectin, fibronectin, vascular endothelial growth factor (VEGF), platelet-derived endothelial growth factor (PDEGF), vitronectin and fibrinogen (coagulation factor II) [14,15,16,17,18].

Animal model studies have demonstrated that in rats receiving bisphosphonates, prior to tooth extraction, local application of VEGF (presented in PRF) to the extraction socket prevents the development of necrotic changes typical of MRONJ. VEGF induces increased expression of genes encoding other proangiogenic factors, such as vWF, VEGF-R2 and CD105, which play a role in tissue healing [9].

Currently, PRF is widely used in medicine, and various fractions are applied in clinical practice, including:

L-PRF (leukocyte PRF), which has a gel form;

A-PRF (advanced PRF), which exhibits a sustained release of transforming growth factor β1 (TGFβ1); PDGF-AB; VEGF; and thrombospondin-1 (TSP-1), (the most important glycoprotein of the coagulation cascade); for at least 7 days [19];

A-PRF+ (advanced PRF plus), characterized by a higher concentration of cells and growth factors;

i-PRF (injectable PRF), a liquid form that enables injection;

T-PRF (titanium PRF), obtained using titanium tubes;

H-PRF (horizontal PRF), allowing for a more homogeneous distribution of cells;

Alb-PRF (albumin PRF), combined with albumin, which prolongs biodegradation time.

Numerous studies have demonstrated that the use of PRF accelerates oral tissue healing by promoting angiogenesis, cell proliferation and tissue regeneration while simultaneously improving patients’ quality of life through a reduction in postoperative pain and discomfort following surgical procedures [20,21,22]. PRF also influences osteoblast proliferation, which may play an important role in the process of bone regeneration [23]. Among studies investigating the use of PRF in the treatment of MRONJ case reports predominate, whereas prospective studies are scarce. The present study is one of the few prospective clinical analyses comparing outcomes of surgical treatment with or without the use of PRF. Unlike most previous studies, this work represents an attempt at a comprehensive evaluation of additional factors that may influence the treatment process, thereby conferring an innovative character to the study.

## 2. Results and Discussion

### 2.1. Results

#### 2.1.1. Patient Characteristics

**Figure 1:**
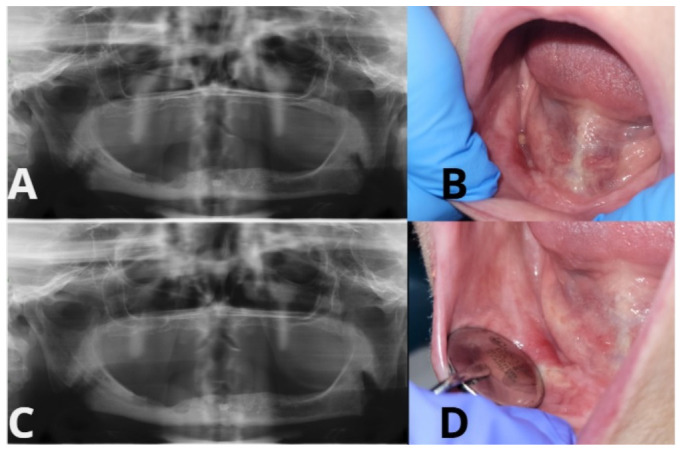
Representative clinical images of surgical sites treated with and without PRF-Patient 18. (**A**) Preoperative OPG. (**B**) Six-week postoperative mucosal healing. (**C**) OPG 6 months after surgery. (**D**) Six-month postoperative mucosal healing.

**Figure 2:**
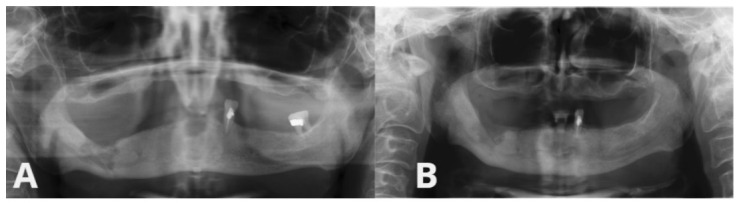
Radiological assessment of Patient 4. (**A**) Preoperative orthopantomogram (OPG) showing MRONJ lesion. (**B**) OPG 6 months after surgical intervention and PRF application, showing radiological improvement.

**Figure 3:**
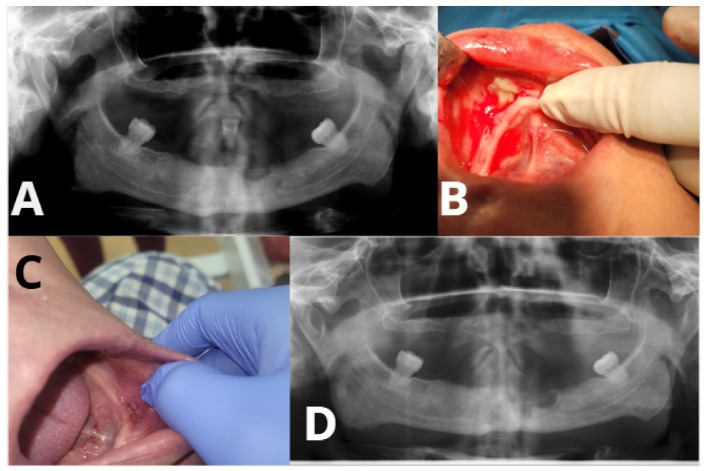
Representative clinical images of surgical sites treated with and without PRF-Patient-Patient 22. (**A**) Preoperative OPG. (**B**) PRF application during procedure. (**C**) OPG 6 months after surgery. (**D**) Six-month postoperative mucosal healing showing granulation tissue without bone exposure.

**Figure 4:**
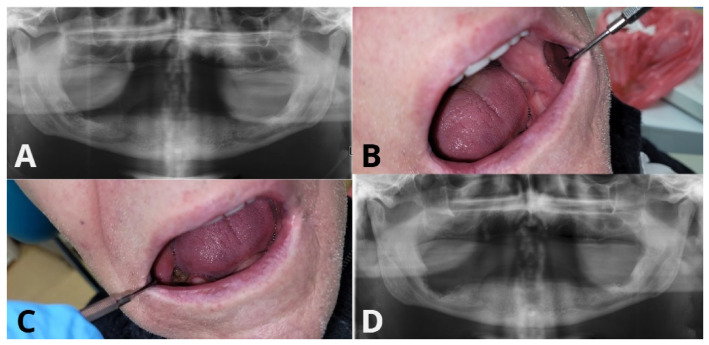
Representative clinical images of surgical sites treated with and without PRF-Patient-Patient 6. (**A**) Preoperative OPG. (**B**,**C**) Six-week postoperative mucosal healing—left: healed; right side—wound dehiscence. (**D**) OPG 6 weeks after surgery.

A total of 22 patients were included in the study. Ten patients were treated with the use of PRF, while in 12 patients, platelet-rich fibrin was not applied. In the PRF group, one patient presented with two MRONJ lesions. Both lesions differed in their clinical presentation and were treated with the use of PRF. All three follow-up time points were completed by 18 patients. Two patients died before completion of the full follow-up period. Several patients failed to attend follow-up visits at the scheduled time points or discontinued follow-up altogether. The mean age of patients in the control group was 63 years, whereas in the PRF group, it was 68.7 years (*p* > 0.05). The number of women was higher in both groups. Smokers constituted a minority of the study population. The most frequent location of lesions was the mandible. Quality of life at admission was higher in patients from the PRF group. The median pain score (NRS scale) at admission was 3 in the control group and 6 in the PRF group, and this difference was statistically significant (*p* < 0.05). There were no serious adverse events during the study. Figure 1, Figure 2, Figure 3 and Figure 4 illustrate radiographs and clinical photographs demonstrating the healing effects of PRF application. Data are presented in Table 1 and Table 2.

All results should be interpreted with caution due to the limited sample size.

#### 2.1.2. Pain Assessment

**Figure 5:**
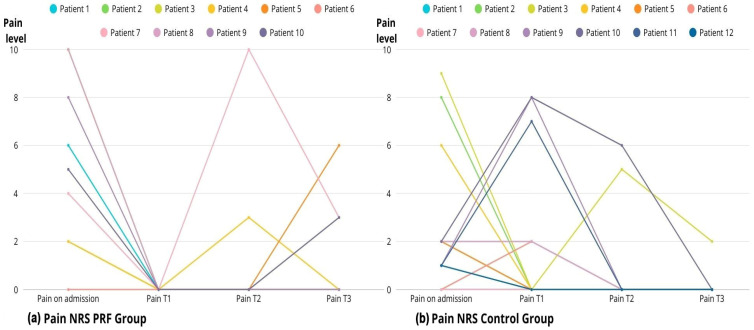
Changes in pain intensity (NRS) over time in PRF (**a**) and control groups (**b**).

A significant reduction in pain was observed postoperatively in both groups (Friedman test, *p* < 0.05). Patients treated with PRF showed a marked reduction in pain as early as the first follow-up visit, which persisted until the final follow-up time point. Pain levels before and after PRF treatment differed significantly, with lower postoperative pain compared to the control group (Wilcoxon test, *p* < 0.05). No comparable reduction in pain was observed in the control group (Wilcoxon test, *p* > 0.05). No significant difference in pain levels was found between the groups at corresponding time points (Mann–Whitney test, *p* > 0.05). (Figure 5a,b; Table 3). It should be noted that baseline pain scores differed significantly between groups (median NRS 6 vs. 3 for the PRF and control groups, respectively; *p* < 0.05), which may affect the interpretation of postoperative pain reduction.

#### 2.1.3. Mucous Integrity Assessment

Improvement in local tissue conditions was observed in the majority of patients. At 14 days postoperatively, only one patient in the study group and three patients in the control group exhibited significant wound dehiscence. The remaining patients showed either complete wound healing or only minor dehiscence. At the T2 visit, the majority of patients did not present with intraoral fistulas. The application of PRF contributed to a reduction in the number of fistulas at the final follow-up; however, this difference compared to the control group was not statistically significant (Table 2).

#### 2.1.4. Quality of Life

QoL did not show a significant improvement in either the control or the study group despite the reduction in pain (Mann–Whitney test, *p* > 0.05). A gradual trend in patient quality of life was observed across the follow-up visits.

#### 2.1.5. AAOMS Stage

**Figure 6:**
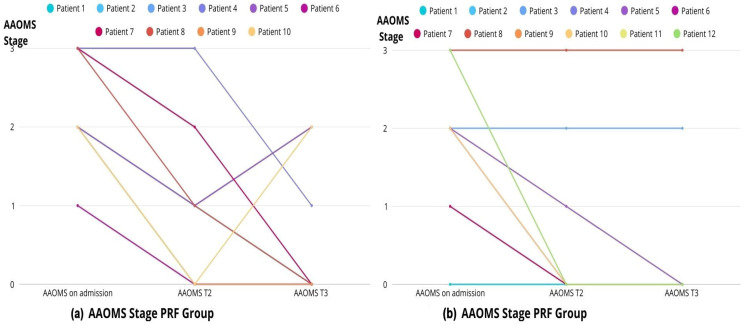
Comparison of AAOMS Stage over time between PRF (**a**) and control groups (**b**).

A significant reduction in AAOMS stage was observed in both groups, with the most pronounced improvement occurring at 6 weeks postoperatively (Friedman test, *p* < 0.05). Similar results were obtained in the pairwise analysis using the Wilcoxon test. The results were comparable between the two groups (Figure 6a,b, Table 4).

#### 2.1.6. Other Factors

A weaker response to PRF treatment was observed in patients with high BMI in terms of AAOMS stage. A negative correlation was found, indicating that as BMI increased, treatment response worsened (Spearman’s rho = −0.646, *p* = 0.044). No such correlation was observed in the control group.

In the control group, patients with elevated CRP concentration exhibited significantly smaller improvements in AAOMS stage.

In contrast, among patients treated with PRF, a reduction (improvement) in AAOMS stage occurred regardless of CRP concentration; neither normal nor elevated CRP values influenced disease severity assessed at 6 weeks and 6 months (Kruskal–Wallis test, *p* < 0.05) (Table 5). PRF treatment in patients with a normal CRP concentration resulted in a pronounced reduction in pain observable from the first follow-up visit and persisting at 6 months. In the control group with normal CRP, changes in pain were less pronounced and did not reach statistical significance in paired time-point analyses.

### 2.2. Discussion

With increasing life expectancy, the number of patients receiving antiresorptive medications for oncological conditions is rising, which correlates with a higher incidence of medication-related osteonecrosis [24,25,26]. Since no “gold-standard” treatment for jaw osteonecrosis has been established, the present study provides additional valuable data regarding the potential use of PRF in the management of MRONJ. The division of patients into groups and the mean age (65.6 years) are consistent with observations reported in other studies supporting the representativeness of the cohort and enabling comparative analyses [3,26,27,28].

One of the main goals of osteonecrosis treatment is pain reduction [3]. In a previous randomized study [29], a significant decrease in pain was observed after PRF application in patients with MRONJ. Similar effects have also been reported in the treatment of both MRONJ and osteoradionecrosis [19]. In the present analysis, patients treated with PRF showed a marked reduction in pain that was significantly greater than in the control group. Importantly, patients in the PRF group initially reported higher pain levels at baseline. A pronounced reduction in pain perception may result from the presence of numerous cytokines within PRF, which modulate nociception. The mechanism of PRF action described in the literature includes the downregulation of interleukin 6 (IL-6), a key mediator involved in the amplification of pain [30,31].

Platelet-rich fibrin contains cytokines critical for tissue regeneration, including:

PDGF (platelet-derived growth factor), which stimulates angiogenesis as well as fibroblast migration and proliferation, contributing to scar formation;

TGF-β (transforming growth factor beta), functioning as a chemotactic factor for leukocytes and fibroblasts;

VEGF (vascular endothelial growth factor), responsible for promoting angiogenesis [32].

Delivery of these factors to the surgical site aims to support reparative processes and accelerate tissue healing. Experimental studies have demonstrated that the administration of vascular endothelial growth factor (VEGF) is associated with enhanced tissue vascularization, which promotes wound healing [33]. Moreover, VEGF has been shown to stimulate osteoblast proliferation and support new bone formation at the site of application [34]. Recent evidence further highlights the role of VEGF in regulating vascular dynamics and tissue regeneration, emphasizing its importance in the healing process [33]. This is particularly important in MRONJ, where the regenerative potential of bone is markedly reduced [20]. These observations are supported by studies evaluating PRF in various oral and maxillofacial surgical and dental procedures [10,35,36]. The potential contribution of a placebo effect associated with patient expectations cannot be entirely excluded [37]. Moreover, the surgical procedure itself in patients with medication-related necrosis may contribute to improvements in pain symptoms [38]. However, the marked reduction in pain observed in the PRF-treated group, along with the modest pain decrease in the control group, appears not to be attributable solely to expectancy effects.

The observed differences in pain reduction between groups should be interpreted with caution, as baseline pain scores were significantly higher in the PRF group, which may introduce bias in the comparison.

A reduction in pain perception is generally associated with an improvement in quality of life; however, the lack of such an effect in the present study may be explained by the progressive nature of the chronic disease, which naturally leads to a decline in QoL [39,40].

The observed decrease in QoL in both groups may also be attributed to the presence of an additional disease, MRONJ, which can independently impact patients’ overall well-being and daily functioning. Furthermore, the potential impact of cancer progression or other comorbid conditions on patients’ perceived quality of life cannot be excluded. It should also be noted that QoL assessment is inherently subjective and individual. Non-medical factors such as deterioration of socioeconomic status or social issues may also influence the reported outcomes [41].

Quality of life in oncology patients is a crucial component of any therapeutic approach [42]. Maintaining QoL at the highest possible level should be an integral part of both treatment and cancer prevention strategies [43]. In the present analysis, patients completed the EQ-5D Visual Analog Scale (EQ-VAS). Both groups initially demonstrated reduced QoL, which showed slight improvement at 6 weeks but declined again by 6 months. Although these differences did not reach statistical significance, they were noticeable.

The absence of exposed bone, indicating mucosal integrity, represents a clinical marker of proper wound healing and consequently improved patient comfort. Postoperatively, improvement in mucosal integrity was observed in both groups and persisted through subsequent follow-up visits. PRF application resulted in slightly better outcomes; however, these differences were not statistically significant. The lack of significance may be attributed to the small sample size.

Enhancement of wound healing achieved through PRF application may reduce the number of required follow-up visits, which is particularly relevant for patients often burdened with multiple comorbidities.

Obesity is a well-documented risk factor for delayed wound healing [44]. This mechanism is primarily associated with metabolic dysfunction of adipose tissue, which, in obesity, becomes an active source of proinflammatory cytokines and adipokines such as Tumor Necrosis Factor Alpha (TNF-α), IL-6 and leptin [45]. These substances promote the persistence of inflammation, disrupt angiogenesis, and impair fibroblast proliferation, ultimately leading to compromised regenerative processes.

In the context of PRF application, this may explain the weaker response observed in patients with elevated body mass index (BMI) compared to individuals with normal body weight. The reduced quality of the healing environment in tissues of obese patients may limit the effectiveness of growth factors released from PRF, negatively affecting tissue regeneration and the rate of wound epithelialization.

The associations related to CRP are also clinically relevant, and its values are frequently elevated in patients with MRONJ [46]. This is likely related to the inflammatory basis of necrosis development [3,4,10,13]. Additionally, in one study, higher CRP concentrations were identified as a marker of postoperative complications [47]. In the present analysis, elevated CRP did not impair wound healing when PRF was applied. Patients achieved better outcomes in terms of AAOMS stage improvement. Such an effect was not observed in patients treated without PRF. Furthermore, PRF application resulted in greater pain reduction compared to the control group, even in patients with normal CRP concentrations, and this effect persisted for up to 6 months. This pattern was not observed in the control group. The results of the analysis indicate poorer wound healing in patients with elevated CRP concentrations. This may result from increased inflammation or involvement of a large area within the oral cavity. The use of PRF partially compensates for the negative effects of inflammation and promotes wound healing. Additionally, several studies have reported a more rapid decrease in inflammatory markers after oral surgical procedures in patients treated with PRF [47,48].

In vivo studies have shown that platelet-rich fibrin reduces levels of inflammatory cytokines and induces a shift in macrophage phenotype from M1 (proinflammatory) to M2 (anti-inflammatory, promoting resolution of inflammation) [49]. Therefore, the application of PRF in patients with elevated CRP may contribute not only to improved wound healing but also to a faster and sustained reduction in pain.

Both BMI and CRP levels may serve as clinically relevant factors influencing treatment outcomes and should be considered in the context of personalized therapeutic approaches. Elevated CRP may reflect an underlying inflammatory status that negatively affects healing, while abnormal BMI values may be associated with impaired regenerative capacity. Incorporating these parameters into clinical decision-making could improve patient stratification and treatment planning.

#### 2.2.1. Strengths and Limitations

This study has several limitations; the primary one is the small number of patients. Additional limitations include the heterogeneity of clinical presentations at the start of treatment and the duration of exposure to medications associated with MRONJ risk. Moreover, it was not possible to verify patient adherence to oral hygiene recommendations or medication regimens. Another limitation of this study is the inability to perform subgroup analyses based on important clinical variables, such as type and duration of antiresorptive therapy, underlying diseases, and concurrent oncological treatment. These factors may influence the development and progression of MRONJ, as well as treatment outcomes [26]. Future studies with larger sample sizes should incorporate these variables to allow for more comprehensive analysis.

A strength of this study is the patient recruitment method, which minimizes the risk of bias and the performance of surgical procedures by different operative teams, not always directly associated with the main investigator. The lack of blinding represents a potential source of bias and should be considered when interpreting the results. Furthermore, the analysis considers additional factors that may influence the course of treatment and that have not previously been evaluated in this way. Many studies are based on case reports or compare patients with MRONJ with those only at risk of MRONJ, which makes comprehensive analysis difficult [50]. We hope that the protocol presented here for comparing study outcomes will support larger analyses and help generate new clinically relevant conclusions for both patients and clinicians.

#### 2.2.2. Further Recommendations

There is a clear need for large, randomized, multicenter studies involving specialists in oral and maxillofacial surgery, dental surgery and oncology. Additionally, the implementation of a standardized patient assessment protocol should be considered, as it would facilitate comparison of study outcomes across different centers. Such a protocol has been developed by our team and is included as an appendix to this study (Supplementary Materials). Its adoption may enable the development of more effective treatment algorithms for patients affected by jaw osteonecrosis.

## 3. Materials and Methods

This study was approved by the Bioethics Committee of the Poznań University of Medical Sciences (resolution no. 808/21, 4 November 2021) and conducted in accordance with the Declaration of Helsinki (revised 2013). The study was retrospectively registered at ClinicalTrials.gov (NCT07464678). Written informed consent was obtained from all participants.

Patients treated for MRONJ at the Department of Oral and Maxillofacial Surgery of the University Clinical Hospital in Poznan between 2022 and 2024 were enrolled in the study. Prior to the initiation of patient recruitment, 50 sequentially numbered, opaque envelopes were prepared by an independent individual not involved in patient recruitment, treatment or outcome assessment. Each envelope contained a card indicating either “PRF” or “Control.” After eligibility confirmation and inclusion in the study, each participant was assigned to the next envelope in sequence, which was then opened to determine group allocation. This procedure ensured allocation concealment and minimized the risk of selection bias. Flow diagram in Supplementary Materials. Due to the surgical nature of the intervention, blinding of both patients and surgeons was not feasible, as they were aware of the treatment performed. However, the use of concealed allocation aimed to reduce potential bias in group assignment. The division into a control group and a group treated with the use of PRF was necessary to compare the effectiveness of both therapeutic approaches and to enable verification of the research hypothesis that the use of PRF improves the outcomes of MRONJ treatment. Patients participated in the study after providing informed consent and met the diagnostic criteria for MRONJ. Patients with metastatic malignant disease affecting the jaw bones, as well as those with hematological disorders such as thalassemia or leukemia, were excluded to avoid confounding factors that could influence the assessment of treatment outcomes. All patients were instructed to report any adverse events or side effects experienced during the study. Potential side effects included infection at the puncture site, significant bleeding, and marked edema.

Before the surgical procedure, the stage of MRONJ was assessed according to the AAOMS classification; pain intensity was evaluated using the Numerical Rating Scale (NRS; 0–10, where 0 indicates no pain, and 10 indicates unbearable pain); and quality of life (QoL) was assessed using an EQ-5D Visual Analog Scale (EQ-VAS) questionnaire (0–100), where 0 represents the worst health imaginable, and 100 represents the best health imaginable. In all patients, laboratory parameters were also assessed, including complete blood count (automated hematology analyzer—Sysmex 1000, Sysmex company, Kobe, Japan), C-reactive protein (CRP) and total protein concentration (standard automated biochemical analyzer—Alinity c, Abbott Company, Abbott Park, IL, USA), and a radiological examination (panoramic radiograph or Computed Tomography) was performed. Follow-up time points were established as follows:

T1—14 days (suture removal): Wound integrity, presence or absence of infection and pain were assessed;

T2—6 weeks: Wound integrity, presence or absence of infection, pain, QoL, radiological assessment and AAOMS stage were evaluated;

T3—6 months: Wound integrity, presence or absence of infection, pain, QoL, radiological assessment and AAOMS stage were evaluated.

T3 was the final study time point; however, patients remained under outpatient follow-up, during which the above-mentioned parameters were routinely assessed.

Therapeutic success was defined as the absence of clinical signs of necrosis at the last follow-up visit, conducted 6 months after the surgical procedure.

The aim of this study was to evaluate the impact of PRF application on selected clinical parameters at individual follow-up stages, including pain intensity, mucosal integrity, presence of infection, quality of life and the stage of necrosis. In addition, the influence of selected systemic factors, such as CRP concentration and body mass index (BMI), on the effectiveness of PRF-based therapy was assessed. Baseline CRP values were classified as normal (<5 mg/dL) or elevated (>5 mg/dL). The collected data were subjected to statistical analysis using Statistica 13.0 (StatSoft, Dell, Round Rock, TX, USA) and the R software (version 4.3.2) with RStudio (version 2023.12.1 + 402; Posit PBC, Boston, MA, USA). Despite the limited sample size, the level of statistical significance was set at *p* < 0.05. Depending on data distribution, assessed using the Shapiro–Wilk test, either parametric or nonparametric tests were applied accordingly.

### Surgical Protocol

Qualification for surgery was based on the decision of a qualified specialist in maxillofacial surgery. During the procedure, a mucoperiosteal flap was elevated to expose the necrotic lesion. Any fistulas present were excised. Necrotic sequestra or necrotically altered bone were removed. The extent of bone removal was determined based on the presence of bleeding from viable bone. Patients in the study group were treated according to a standardized surgical protocol in which PRF was applied following removal of necrotic tissue. PRF was used in the form of clots and placed directly in the surgical site to fill the residual bone defect. Typically, two PRF clots were applied per patient, depending on the size of the defect. PRF was prepared according to the Choukroun A-PRF+ protocol: 40 mL of autologous venous blood was collected into four 10 mL glass tubes without anticoagulants and immediately centrifuged at 1300 rpm for 14 min using a PRF Duo Quattro centrifuge (Process for PRF, Nice, France) [51]. After placement of PRF, the surgical wound was closed with sutures, ensuring tension-free closure. All patients received antibiotic prophylaxis consisting of amoxicillin with clavulanic acid (875 mg + 125 mg, twice daily) or clindamycin (600 mg, three times daily) in cases of penicillin allergy, administered 12 h prior to surgery. Antibiotic therapy was continued for 14 days postoperatively. When microbiological swab results and an antibiogram were available, antibiotic therapy was modified according to the antibiogram. Postoperatively, patients were given instructions regarding proper oral hygiene and were prescribed a chlorhexidine mouth rinse (0.2%). All procedures were performed in a standardized manner by surgical teams consisting of a specialist and a resident or two specialists in order to ensure comparability of results.

No patient in the study group reported serious adverse events related to the PRF harvesting procedure. In the postoperative period, patients occasionally reported bruising at the venipuncture site, mild swelling or minimal pain. Patient follow-up was conducted in the outpatient clinic according to a predefined standardized protocol, including follow-up visits at 14 days, 6 weeks, and 6 months after surgery. During each visit, clinical and radiological parameters were assessed, including the soft tissue healing, presence or absence of bone exposure, pain complaints, AAOMS stage, quality of life, presence of exudate and radiological findings. Assessments were performed by different members of the clinical team in order to limit the subjective influence of a single observer and to increase the reliability of the obtained results. Improvement in patient condition was indicated by a reduction in AAOMS stage, decreased pain intensity and mucosal integrity.

## 4. Conclusions

MRONJ remains a condition for which no definitive standard of treatment has been established. Numerous clinical studies continue to shed light on this relatively recent disease entity. Drawing appropriate conclusions from these studies allows for better planning of future research and the identification of effective treatment strategies. The use of platelet-rich fibrin is a safe and cost-effective procedure. Its application may enhance the potential benefits for patients treated for MRONJ. The risk of adverse effects associated with PRF is minimal. Target populations may particularly include patients experiencing severe pain as well as those with pronounced clinical signs of inflammation and elevated inflammatory parameters. This study should be considered a pilot study due to the limited sample size. Therefore, the findings should be interpreted with caution.

Further research is needed, especially multicenter studies and meta-analyses. To date, only a limited number of prospective clinical studies have evaluated the use of PRF in this setting. We also did not identify any studies that consider factors such as CRP, BMI or QoL in the treatment of MRONJ patients with PRF. We hope that the results presented here will encourage further research design and serve as a basis for future meta-analyses.

## Figures and Tables

**Table 1:** Baseline characteristics of the study population. Legend: PRF—platelet-rich fibrin, W—women, M—men, BMI—body mass index, SD—standard deviation, NRS—Numeric Rating Scale, QoL—quality of life, CRP—C-reactive protein.

Parameter	Control	PRF	*p*-Value
Number of patients	12	10	
Gender W/M	8/4	7/3	
Age [years]	63 [SD11.85]	68.7 [SD 9.18]	>0.05
BMI [kg/m^2^]	25.4 [SD 3.94]	24.23 [SD 3.69]	>0.05
Number of smokers	3	1	
Location in mandible	11	8	>0.05
Intraoral fistula on admission	11	11	>0.05
Pain on admission [median NRS]	3	6	<0.05
QoL on admission	58.64 [SD 25.41]	78 [SD 23.12]	>0.05
CRP on admission [mg/dL]	11.36 [SD 16.48]	3.65 [SD 3.84]	>0.05
Total protein [mg/dL]	6.87 [SD 0.44]	6.61 [SD 0.44]	>0.05

**Table 2:** Clinical characteristics of MRONJ lesions and treatment details. Legend: PRF—platelet-rich fibrin. ( )—total number on follow-up, QoL—quality of life, SD—standard deviation, AAOMS—American Association of Oral and Maxillofacial Surgeons, PRF—platelet-rich fibrin.

Parameter	Control	PRF	*p*-Value
Major wound dehiscence or complete lack of healing	3(12)	1(10)	>0.05
Pain level on 1st follow-up [median]	0	0	>0.05
Improvement of pain level on 1st follow-up	7(12)	9(10)	>0.05
Pain level on 2nd follow-up [median]	0	0	>0.05
QoL on 2nd follow-up	63.13 SD [18.7]	78,33 SD [21.51]	>0.05
Improvement of QoL on 2nd follow-up	3(8)	3(8)	>0.05
Improvement of AAOMS level on 2nd follow-up	6(8)	10(10)	>0.05
Pain level on 3rd follow-up [median]	0	0	>0.05
QoL on 3rd follow-up	46 SD [23.29]	69.25 [SD20.91]	>0.05

**Table 3:** Comparison of postoperative outcomes between PRF and control groups. Legend: PRF—platelet-rich fibrin.

Wilcoxon Test	PRF		Control	
	Pain reduction median		Pain reduction median	
Pain T1 vs. before surgery	5.5	*p* < 0.05	2	*p* > 0.05
Pain T3 vs. before surgery	3	*p* < 0.05	2	*p* > 0.05

**Table 4:** Analysis of factors influencing wound healing outcomes Legend: PRF—platelet-rich fibrin, AAOMS—American Association of Oral and Maxillofacial Surgeons.

Wilcoxon Test	PRF		Control	
Improvement AAOMS T2 vs. before surgery	YES	*p* < 0.05	YES	*p* < 0.05
Improvement AAOMS T3 vs. before surgery	YES	*p* < 0.05	YES	*p* < 0.05

**Table 5:** Changes in pain intensity (NRS) during follow-up Kruskal–Wallis test AAOMS improvement, 6 weeks. Legend: AAOMS—American Association of Oral and Maxillofacial Surgeons; CRP—C-reactive protein; PRF—platelet-rich fibrin.

AAOMS Improvement at 6 Weeks	Kruskal–Wallis Test; AAOMS Improvement at 6 Weeks According to CRP Concentration Kruskal–Wallis Test: H (3, N = 19) = 11.66667 *p* = 0.0086			
	Code	N	Rank Sum	Mean Rank
Control + normal CRP	1	6	69	11.50
PRF + normal CRP	2	9	94	10.44
Control + elevated CRP	3	2	4	2
PRF + elevated CRP	4	2	23	11.50

## Data Availability

The datasets generated and analyzed during the current study are available from the corresponding author upon reasonable request. All patients provided consent for the publication of photographs and radiographic images. All presented data are anonymized.

## Supplementary Materials

The following supporting information can be downloaded at https://www.mdpi.com/article/10.3390/ijms27083654/s1.

## Abbreviations

A-PRF	Advanced Platelet-Rich Fibrin
A-PRF+	Advanced Platelet-Rich Fibrin Plus
AAOMS	American Association of Oral and Maxillofacial Surgeons
Alb-PRF	Albumin Platelet-Rich Fibrin
BMI	Body Mass Index
BRONJ	Bisphosphonate-Related Osteonecrosis of the Jaw
CRP	C-Reactive Protein
H-PRF	Horizontal Platelet-Rich Fibrin
HIV	Human Immunodeficiency Virus
I-PRF	Injectable Platelet-Rich Fibrin
IL-6	Interleukin 6
L-PRF	Leukocyte Platelet-Rich Fibrin
M	Men
MRONJ	Medication-Related Osteonecrosis of the Jaw
NRS	Numeric Rating Scale
QoL	Quality of Life
PDEGF	Platelet-Derived Endothelial Growth Factor
PRF	Platelet-Rich Fibrin
SD	Standard Deviation
T-PRF	Titanium Platelet-Rich Fibrin
TGFβ1	Transforming Growth Factor Beta
TNF- α	Tumor Necrosis Factor alpha
TSP-1	Thrombospondin 1
VEGF	Vascular Endothelial Growth Factor
vWF	von Willebrand Factor
W	Women
