# Supplementary material for: Platelet-Rich Fibrin in Surgical Wound Healing of Medication-Related Osteonecrosis of the Jaw: A Pilot Clinical Study
Source: Int J Mol Sci. 2026 Apr 20;27(8):3654. doi: 10.3390/ijms27083654 (PMC13116275; doi:10.3390/ijms27083654)
Supplement: Supplementary file 1 [file ijms-27-03654-s001.zip › ijms-4250620.pdf]

Assesement Protocol for MRONJ Patient

Date

Name

Surname

ID and age

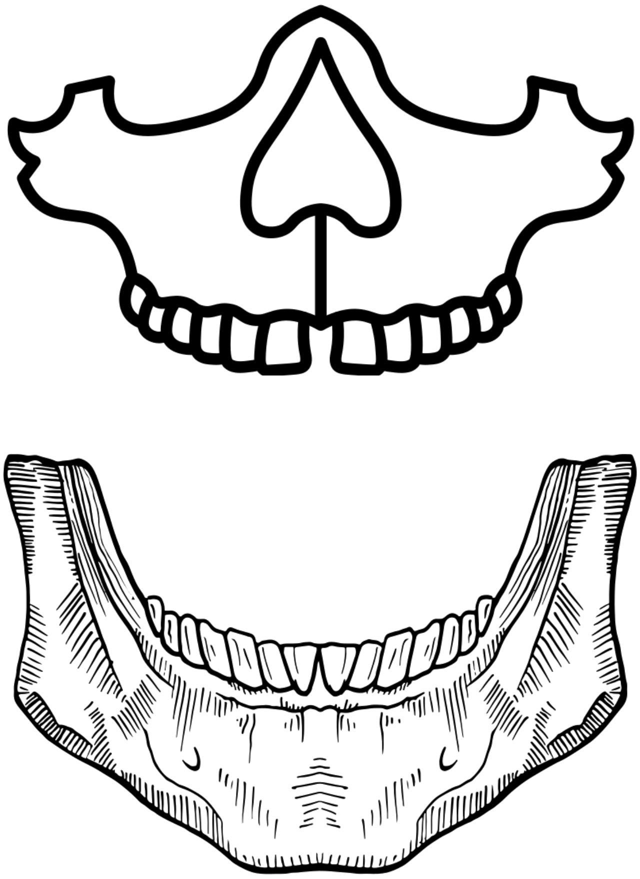

Location

MRONJ Data

Potential Drug(s)

Time and route of administration

First symptoms

Date of diagnosis

Clinical Assesment

Stage AAOMS

Pain NRS

Intraoral fistula

Extraoral fistula

Laboratory results

CRP

WBC

RBC

Others (eg. QoL)

Improvement/worsening compared to the previous visit:

Treatment

Conservative

Surgery

Other (eg. PRF,

Hyperbaric oxygen)

Else

Smoking status

Diseases

BMI
