# Supplementary figures and images for: Platelet-Rich Fibrin in Surgical Wound Healing of Medication-Related Osteonecrosis of the Jaw: A Pilot Clinical Study
Source: Int J Mol Sci. 2026 Apr 20;27(8):3654. doi: 10.3390/ijms27083654 (PMC13116275; doi:10.3390/ijms27083654)

## Flow Diagram

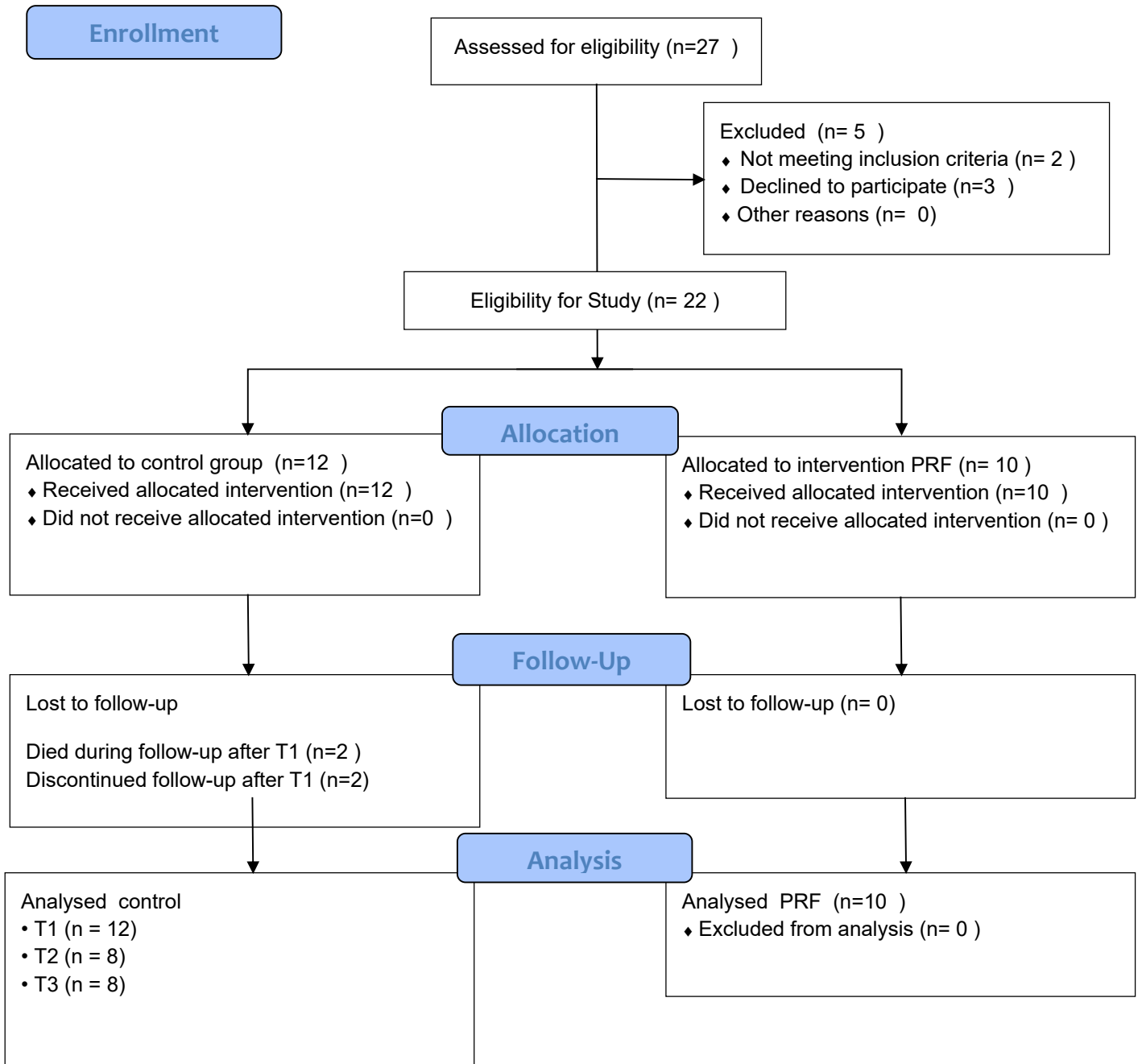

Supplement: Supplementary file 1 [file ijms-27-03654-s001.zip › ijms-4250620-supplementary.pdf]
